# Nanogram-Scale Preparation and NMR Analysis for Mass-Limited Small Volatile Compounds

**DOI:** 10.1371/journal.pone.0018178

**Published:** 2011-03-28

**Authors:** Satoshi Nojima, David J. Kiemle, Francis X. Webster, Charles S. Apperson, Coby Schal

**Affiliations:** 1 Department of Entomology and W. M. Keck Center for Behavioral Biology, North Carolina State University, Raleigh, North Carolina, United States of America; 2 Department of Chemistry, State University of New York-Environmental Science and Forestry, Syracuse, New York, United States of America; University of California Davis, United States of America

## Abstract

Semiochemicals are often produced in infinitesimally small quantities, so their isolation requires large amounts of starting material, not only requiring significant effort in sample preparation, but also resulting in a complex mixture of compounds from which the bioactive compound needs to be purified and identified. Often, compounds cannot be unambiguously identified by their mass spectra alone, and NMR analysis is required for absolute chemical identification, further exacerbating the situation because NMR is relatively insensitive and requires large amounts of pure analyte, generally more than several micrograms. We developed an integrated approach for purification and NMR analysis of <1 µg of material. Collections from high performance preparative gas-chromatography are directly eluted with minimal NMR solvent into capillary NMR tubes. With this technique, ^1^H-NMR spectra were obtained on 50 ng of geranyl acetate, which served as a model compound, and reasonable H-H COSY NMR spectra were obtained from 250 ng of geranyl acetate. This simple off-line integration of preparative GC and NMR will facilitate the purification and chemical identification of novel volatile compounds, such as insect pheromones and other semiochemicals, which occur in minute (sub-nanogram), and often limited, quantities.

## Introduction

NMR spectrometry is a powerful tool to identify the chemical structures of novel compounds. However, in order to acquire high quality spectra for structure elucidation, the NMR method requires relatively large amounts of pure compound (up to 100 µg), often requiring substantial purification from extracts or other mixtures of compounds. The requirement for large amounts of pure material comes from the inherent relatively low sensitivity of NMR and the lack of a compound separation system in NMR analysis. This severely limits the usefulness of NMR when the source or amount of biological material is limited.

To overcome these drawbacks of NMR analysis, special probes and instruments have recently been developed [1], [2], [3], [4], [5], [6], and these techniques have pushed the lower boundary of NMR analysis into the microgram sample scale in some cases. In recent years, highly sensitive capillary NMR techniques that combine cryogenic stages and capillary NMR tubes have become popular [5], [7]. Notably, highly specialized, microcoil probes with extremely low active volumes have also been developed, but these NMR approaches require microfabrication and are not in routine use [6], [8].

Although hyphenated NMR techniques, such as online LC-NMR, CE-NMR, and even GC-NMR have been developed to overcome the lack of separation systems in NMR analysis [5], [7], [9], [10], the application of these NMR techniques remains challenging, particularly for unknown small volatile compounds at sub-microgram amounts, when they occur in limited supply in a complex matrix of other compounds, as is typical in many projects involving semiochemicals.

Preparative-GC is a very useful, and perhaps the only effective means to efficiently purify volatile compounds at a substantial level for NMR analysis [11], [12], [13], [14], [15]. We have developed an easy-to-construct, easy-to-operate, high performance preparative GC system [16]. Compound separation and collection is highly efficient in the mass range of a few nanograms to several micrograms per operation with recovery efficiencies >85% and without any significant contamination. This system makes it easy to isolate small volatile compounds from complex matrices.

We describe here a simple off-line integration of the preparative GC technique and capillary NMR analysis to facilitate the identification of minute amounts of small volatile compounds.

## Methods

### Materials

Geranyl acetate (Aldrich, MW 196) was used as a model volatile compound. Benzene-d_6_ (99.96% deuteration) was purchased from Cambridge Isotope Laboratories.

### Preparative Gas-Chromatography

An HP5890 GC, modified as a preparative GC system according to [16], was used in this study. A split-splitless (S/SL) injection port assembly was installed adjacent to an FID port and modified as a sample collection port. The heater and sensor of the assembly were connected to the control ports assigned to detector B so that the temperature of the collection port could be controlled and monitored as “detector B”. A direct injection glass liner (1 mm Uniliner® for 0.32 and 0.53 mm ID columns, 1.0 mm ID, 6.3 mm OD ×78.5 mm, Restek) was installed in the collection port as an interface adaptor between a separation column and the collection traps. A splitter, which was connected to the end of a separation column, was composed of two deactivated transfer lines (a 35 cm section of 0.53 mm ID and a 70 cm length of 0.25 mm ID, no stationary phase; Grace Davison) and a universal Y connector (Grace Davison). The megabore, 0.53 mm ID transfer line, was connected to the interface adaptor in the collection port, while the 0.25 mm ID transfer line was connected to the FID. The split ratio was about 1:25 under this specification.

Either a non-polar EC-5 or polar EC-WAX megabore capillary column (1.0 µm film thickness, 0.53 mm ID ×30 m, Grace Davison) was used as separation column. Helium was used as carrier gas at a head pressure of 27.5 kPa and at a flow rate of 6.8 ml/min. The oven temperature was set at 50°C for 2 min, increased at 15°C/min to 250°C, and held for 5 min. The inlet temperature was set at 270°C, and the collection port temperature was held at 250°C. No make-up gas was used for the FID, because the carrier gas flow and pressure of the 0.25 mm ID transfer line were too low to maintain flow to the FID against the back-pressure of the FID make-up gas. Good FID recordings were obtained, nonetheless, without the make-up gas.

**Figure 1:**
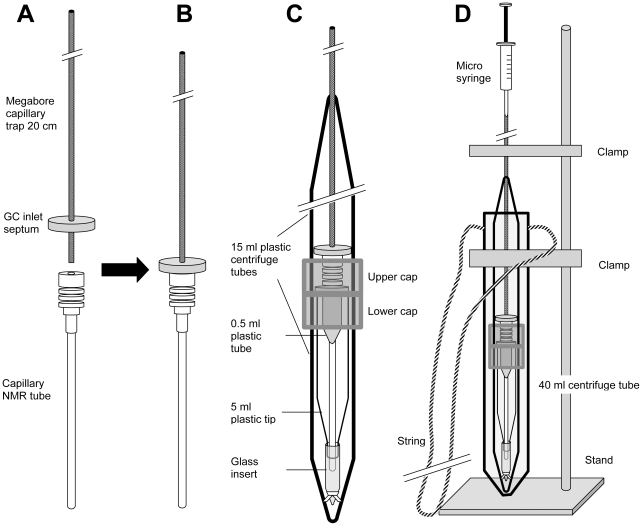
a–d Schematic diagrams of assembly holder for NMR sample recovery and preparation.

Sections (20 cm) of megabore capillary columns (DB-1, 0.53 mm ID, 1 or 5 µm film thickness; Agilent) were used as collection traps [16]. These collection traps were rinsed twice with 100 µl of dichloromethane (high purity solvent, Honeywell Burdick & Jackson) and dried at room temperature overnight before use. A predrilled GC septum from Supelco was passed through a trap as a retainer stopper for the extraction process of trapped compounds (Fig. 1).

### Assembly Holder for Sample Recovery and NMR Sample Preparation

An assembly holder was made and used for sample recovery and NMR sample preparation (Fig. 1). Two 15 mL centrifuge tubes were used to house and align a capillary NMR tube and a collection trap. The tubes were connected upside down at the top, and fixed with duct tape (Fig. 1c). A 5 mL plastic pipette tip was used as a guide and protector for the capillary NMR tube, while a 0.3 mL glass conical insert (for autosampler vials, Fisher Scientific) was used for safety, in case the NMR tube broke and sample needed to be recovered (Fig. 1c); this never happened in dozens of sample recovery operations. The length of the 5 mL pipette tip was adjusted by cutting off the top portion so that the pipette tip and the glass insert could be snugly housed in the lower centrifuge tube. A hole in the cap of the lower centrifuge tube accommodated a 0.5 mL plastic centrifuge tube whose lid/cap was removed; a hole at the bottom of this tube allowed the capillary NMR tube to pass through. The 0.5 mL tube both securely held the head of the NMR tube and guided the NMR capillary.

A hole in the cap of the inverted upper 15 mL centrifuge tube also accommodated the head of NMR tube, and another hole at the tip of the tube (top of the assembly Fig. 1c) allowed the collection trap to pass through into the NMR tube. The entire assembly was housed in a 40 mL centrifuge plastic tube for manual centrifugation (Fig. 1d). This larger holder was made from two 40 mL plastic centrifuge tubes. A loop of strong string was fixed at the lip of the tube, and the holder was held on a lab stand by a clamp (Fig. 1d).

### Sample Purification, Collection, and Preparation for NMR Analysis

The general procedure of preparative GC purification and sample collection are according to Nojima et al. [16]. Geranyl acetate was injected (in hexane) into the preparative GC, separated from the solvent and other impurities on the GC column, and collected from the collection port. The collection trap was pressed into the adaptor at the collection port just before geranyl acetate eluted. By gently pressing the trap against the port a secure tight seal was achieved between the port and a trap. At the end of the collection window, the trap was pulled out from the port. Collections were made at ambient temperature without cryogenic treatment. In a preliminary experiment, the recovery efficiency of geranyl acetate under this collection condition was almost 100%, as expected from our previous study [16].

### Sample Recovery and NMR Sample Preparation

The entire assembly for sample recovery and NMR sample preparation was setup on a lab stand (Fig. 1d). The upper 15 mL plastic centrifuge tube was removed (unscrewed from its cap), and a capillary NMR tube (Bruker BioSpin) was placed through the hole in the cap into the lower tube. The retainer (GC septum) was adjusted to about 5 mm above the end of the collection trap that was in the collection port (Fig 1a). This prevented the collection trap from being damaged within the NMR tube during the manual centrifugation. Then, the collection trap was set up on a capillary NMR tube (Fig. 1b, c and d), and the upper centrifuge tube was screwed on the upper cap. We injected 7–8 µl of benzene-d6 into the top of the collection trap using a GC syringe equipped with a 23 gauge cone tip needle (Fig. 1d). The NMR solvent remained at the bottom end of the collection trap due to capillary phenomena. Immediately after solvent injection, the whole assembly was removed from the stand and swung by hand using the string for 1 min. After the manual centrifugation, the collection trap was removed from the NMR tube, and the opening of the tube was plugged with a Teflon ball (Bruker BioSpin). Sample purification and NMR sample preparation were conducted in a room kept at about 20°C.

### Background Signals

Background NMR signals, which originate from unavoidable contaminants and impurities from various sources, were checked. In order to specify those signals, first, fresh benzene-d6 was loaded into an NMR tube with a GC syringe, and the tube was subjected to ^1^H-NMR analysis as a blank sample.

Background signals of control samples were also checked. Hexane was injected into the preparative GC equipped with either EC-5 or EC-WAX columns, and control collections were made under the same analytical conditions as described above. NMR samples were prepared from these control collections in the same manner as for the model compound, and these NMR samples were subjected to ^1^H-NMR analysis as control samples.

### Detection Limits of NMR Analysis

Various concentrations of geranyl acetate in benzene-d6 were prepared. Five µl of each standard solution were loaded into capillary NMR tubes using a syringe and subjected to ^1^H-NMR analysis. Two dimensional H-H DQF COSY NMR experiment was conducted only on 250 ng sample.

### NMR Spectroscopy

**Figure 2:**
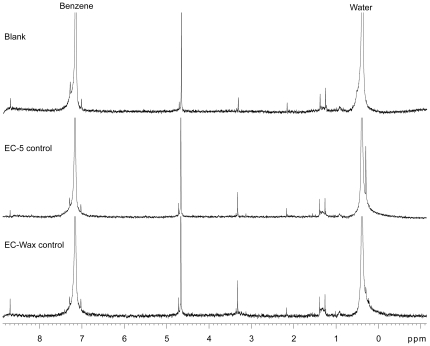
Background signals of blank and control NMR samples.

**Figure 3:**
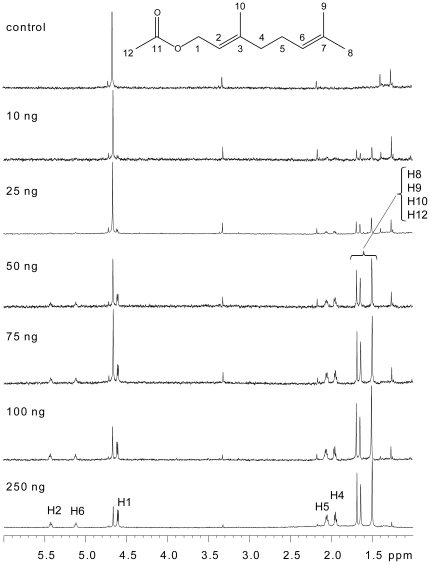
Detection limits of ^1^H-NMR of geranyl acetate.

**Figure 4:**
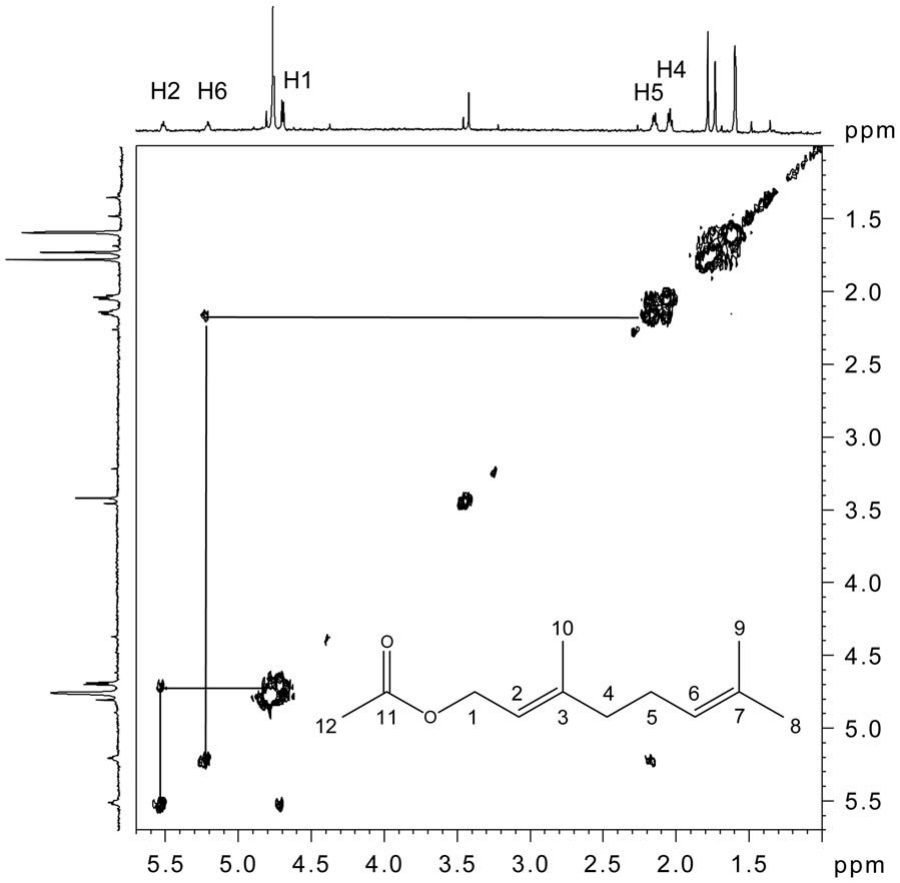
H-H COSY NMR spectrum of 250 ng geranyl acetate.

All spectra were acquired at 25°C with a Bruker AVANCE 600 MHz spectrometer equipped with a 1-mm triple resonance z-gradient microprobe, with spectral widths of 4800 Hz (8.0 ppm). 1-D Proton spectra (Figs. 2 and 3) were acquired with a 4.18 µsec 90° pulse, with a 1.5 sec recycle delay and 1.5 sec acquisition time. Depending on the concentration of the sample, spectra were acquired with 4000 to 8000 scans. Data were processed with exponential decay equivalent to 1 Hz line broadening. The 2-D gradient magnitude calculated COSY spectrum (Fig. 4) was acquired with 2048 data points in F2 by 142 data points in F1, a 4.18 µsec 90° pulse, with a 1.0 sec recycle delay and 0.21 sec acquisition time with a total time of 50 hrs to acquire data. The COSY was processed as a 2k ×256 matrix with a shifted (p) qsin weighting function. Data were symmetrized. The signal of trace amounts of benzene, which is an unavoidable contaminant in NMR samples, was used as a reference.

## Results and Discussion

### Background Signals

It is essential to minimize and specify background signals to establish and achieve sub-microgram scale NMR analysis. However, there are unavoidable contaminants from various sources, such as the NMR tubes, syringes, intrinsic impurities in NMR solvents, and so on. Even minute amounts of contaminants, which are of no concern in routine NMR analysis, can be a significant problem at small sample scale. In order to specify background signals and evaluate their significance for small scale analysis, fresh benzene-d6 was loaded into NMR tubes with a GC syringe, and the sample was subjected to ^1^H-NMR analysis (Fig. 2). The blank benzene-d6 sample showed strong signals from a trace amount of benzene at 7.16 ppm and from water at 0.39 ppm, a modest signal of unknown origin at 4.66 ppm (possibly methanol), and a few minor signals between 1 and 3.5 ppm. There was no significant signal in the lower field. The numbers and intensities of these signals were acceptable in comparison to geranyl acetate low amounts (Fig. 3). In addition, the background signals were separated from those of the model compound. Thus, overall, background signals are not significant for small-scale NMR analysis for geranyl acetate, and likely not for most other compounds.

Chloroform-d is commonly used as an NMR solvent. But this solvent is spontaneously oxidized to phosgene which is easily hydrolyzed to hydrochloric acid, a highly reactive byproduct. Trace amounts of hydrochloric acid can be fatal for NMR samples, especially at low concentrations of analytes that require either long storage or long residence time in the magnet for various NMR experiments. On the other hand, benzene-d6 is a stable, non-reactive compound, and thus it is preferable as an NMR solvent for small samples whose chemical stability is unknown. Also, residual water in chloroform-d often overlaps with important analyte peaks at about 1.5 ppm whereas the residual water peak in benzene-d6 is found at 0.4 ppm and rarely overlaps with important analyte peaks. In a preliminary experiment, background signals of benzene-d6 from various commercial sources were compared, and one from Cambridge Isotope Laboratories, Inc., was found to have the fewest and weakest background signals.

Background signals of control samples were also analyzed (Fig. 2). There are various opportunities to introduce contaminants during the preparative GC purification, sample recovery, and NMR sample preparation steps. For example, solvent impurities and bleeding from the stationary phase of columns can interfere with NMR analysis. In addition, the stationary phase in our collection traps – short sections of megabore column – can bleed into the NMR tubes during elution. The stationary phase is critical for high efficiency of the collection traps in preparative GC, as it makes it possible to trap volatile compounds at room temperature, which is very convenient for multiple fractionations during a single preparative GC run; traps without stationary phase were much less effective [16].

Control preparative GC collections were made from hexane injections on both EC-5 and EC-WAX columns, NMR samples prepared, and subjected to ^1^H-NMR analysis. The NMR spectra of control samples were compared with that of blank sample (Fig. 2). The intensity of the spectra is standardized by the signal at 4.66 ppm which was consistently found in all samples at similar intensity suggesting an intrinsic impurity in benzene-d6. Overall, there was no difference in the ^1^H-NMR profiles between the blank and control samples, except a few minor signals around the large signal from water. These results show that the preparative GC purification step, and later sample recovery and NMR sample preparation steps did not introduce significant contaminations into the NMR tubes. They also suggest that the minor background signals likely come from intrinsic impurities in benzene-d6. The signal-to-noise ratios varied across each spectrum because samples were not run concurrently and sensitivity of the NMR instrument might have been slightly different at each analysis. Nevertheless, it is clear that a major advantage of eluting the collection trap with benzene-d6 directly into the low-volume probe minimized signals from solvent impurities.

### Detection Limits of NMR Analysis

There are no systematic investigations on the detection limits of NMR analysis. Eyres et al. [15] used preparative multidimensional GC to isolate geraniol from an essential mixture and subjected the pure compound to offline NMR analysis; the 5-mm cryoprobe used 8.6 µg geraniol for proton and gradient correlation spectroscopy (gCOSY) experiments and 77.6 µg geraniol for gHSQC and gHMBC NMR experiments. The same preparative GC approach was also used to obtain 3.1 µg and 5.0 µg of 1- and 2-methylnaphthalene, respectively, for ^1^H-NMR spectroscopy [15]. Using the newly-developed 1-mm probe, Schlotterbeck et al. [1] were able to acquire ^1^H^13^C gHSQC 2D NMR spectra of 1 µg of ibuprofen within 20 hrs, and long-range ^1^H^13^C gHMBC spectra of 20 µg of ibuprofen within 5 hrs; they also acquired a ^1^H-NMR spectrum within 14 min of 1 µg of boldine that was fractionated from a 1-mm HPLC column. Finally, Hu et al. [2] showed that using a capillary NMR probe ∼5 µg is needed for one-dimensional proton and two-dimensional homonuclear (COSY and NOESY) NMR experiments, ∼30 µg for HMQC- or HSQC-NMR spectra, between 75 and 100 µg are necessary to measure HMBC-NMR spectra, and ∼200 µg are needed for C-13- and DEPT-NMR experiments. Although much lower detection limits have been achieved with specialized solenoid radio-frequency coils [6], [8], these micro-scale NMR approaches require highly specialized microfabrication or micromachining of NMR microcoil probes.

We directly loaded 1-mm capillary NMR tubes with known amounts of the model compound geranyl acetate, and subjected them to ^1^H-NMR analysis. The results are shown in Fig. 3. There was a good relationship between the mass of geranyl acetate in the tube and signal intensity. Remarkably, under these conditions, distinct singlet proton resonances of the methyl groups of geranyl acetate were observable even at 10 ng (51 pmol/tube), while other coupled signals were distinctively observed between 25 and 50 ng. Reasonable to excellent signals across all ranges were obtained at ≥75 ng sample scale at acceptable run times of 3–18 hrs. It should be noted that the noise levels varied among the samples due to different run times.

Based on these results, we suspected that 250 ng might be the minimum mass for a two dimensional NMR spectrum, H-H COSY, in reasonable run time. Therefore, the H-H COSY experiment was conducted only on 250 ng of geranyl acetate. The spectrum is shown in Fig. 4. The spin networks of protons, H1-H2, H4-H5, H5-H6, were clearly observed in 2 days run time. Using various amounts of geranyl acetate as a model compound, samples were purified by preparative GC and subjected to ^1^H-NMR analysis. The results, over all, were identical to those obtained with the standard samples that were loaded directly into NMR tubes. Thus, simple off-line integration of the preparative GC with capillary NMR analysis is highly efficient for mass-limited small volatile compounds.

### Concluding Remarks and Practical Application

We have established an integrated technique using a high performance preparative GC to purify small amounts of volatile compounds and prepare them for capillary NMR analysis. By minimizing solvent use in preparative GC, sample elution, and sample transfer to the NMR tube, signal-to-noise ratio can be optimized, and the inadvertent introduction of impurities is minimized. Consequently, reliable ^1^H-NMR experiments can be completed on <100 ng of GC-purified samples, while a few hundred ng is required for two dimensional homonuclear NMR analysis. GC sample purification, elution, and transfer to capillary NMR tubes can be performed with readily available materials, and the apparatus, coupled with a high performance preparative GC [16], is easy to construct and easy to operate. This integrated technique is therefore technically and economically feasible for many researchers.

We have used this approach to isolate and identify several insect semiochemicals for which MS results were not sufficient to deduce a structure, with a range of sample amounts from 0.4 to 10 µg. In a few cases, starting with the isolation and NMR analysis of blattellaquinone, the sex pheromone of the German cockroach [13], multiple collections were made using different collection traps, and the trapped compound was eluted into the same NMR tube at every collection, while in recent collections, multiple collections were made in the same trap, and it was kept on a lab bench at room temperature between collections without contamination or significant sample loss. Chemical identifications of a cockroach (*Parcoblatta lata*) sex pheromone and mosquito (*Aedes aegypti*) oviposition-site attractants through use of this integrated approach will be reported elsewhere. For both, ^1^H-NMR and various two dimensional NMR experiments were conducted on <2 µg of GC-purified material.
